# The Antitumor Activity of Sodium Selenite Alone and in Combination with Gemcitabine in Pancreatic Cancer: An In Vitro and In Vivo Study

**DOI:** 10.3390/cancers13133169

**Published:** 2021-06-25

**Authors:** Kevin Doello, Cristina Mesas, Francisco Quiñonero, Gloria Perazzoli, Laura Cabeza, Jose Prados, Consolacion Melguizo, Raul Ortiz

**Affiliations:** 1 Medical Oncology Service, Virgen de las Nieves Hospital, 18014 Granada, Spain; kdoello@correo.ugr.es; 2 Instituto Biosanitario de Granada (ibs. GRANADA), 18014 Granada, Spain; cristinam@correo.ugr.es (C.M.); fjquinonero@ugr.es (F.Q.); gperazzoli@ugr.es (G.P.); lautea@ugr.es (L.C.); melguizo@ugr.es (C.M.); roquesa@ugr.es (R.O.); 3 Center of Biomedical Research (CIBM), Institute of Biopathology and Regenerative Medicine (IBIMER), University of Granada, 18100 Granada, Spain; 4 Department of Anatomy and Embryology, Faculty of Medicine, University of Granada, 18071 Granada, Spain; 5 Department of Medicine, Faculty of Health Sciences, University of Almería, 04120 Granada, Spain

**Keywords:** sodium selenite, gemcitabine, pancreatic cancer, cancer stem cells, combined therapy, phospho-p38 protein

## Abstract

**Simple Summary:**

The treatment of advanced pancreatic cancer represents a major challenge because of its chemoresistance and low survival rate. The aim of this research is to test a new antitumor agent (sodium selenite) in pancreatic cancer, both in vitro and in vivo, focusing on the molecular mechanisms involved and its combination with traditional chemotherapy (gemcitabine). Our results show that selenite has a strong antitumor effect against pancreatic cancer, via parthanatos cell death, and enhances the effect of gemcitabine via the p38 pathway in both in vitro and in vivo assays. These results could improve the outcome of pancreatic cancer patients and could be the start of future clinical trials.

**Abstract:**

Sodium selenite acts by depleting enzymes that protect against cellular oxidative stress. To determine its effect alone or in combination with gemcitabine (GMZ) in pancreatic cancer, we used PANC-1 and Pan02 cell lines and C57BL mice bearing a Pan02-generated tumor. Our results demonstrated a significant inhibition of pancreatic cancer cell viability with the use of sodium selenite alone and a synergistic effect when associated with GMZ. The molecular mechanisms of the antitumor effect of sodium selenite alone involved apoptosis-inducing factor (AIF) and the expression of phospho-p38 in the combined therapy. In addition, sodium selenite alone and in association with GMZ significantly decreased the migration capacity and colony-forming ability, reduced tumor activity in multicellular tumor spheroids (MTS) and decreased sphere formation of cancer stem cells. In vivo studies demonstrated that combined therapy not only inhibited tumor growth (65%) compared to the untreated group but also relative to sodium selenite or GMZ used as monotherapy (up to 40%), increasing mice survival. These results were supported by the analysis of C57BL/6 albino mice bearing a Pan02-generated tumor, using the IVIS system. In conclusion, our results showed that sodium selenite is a potential agent for the improvement in the treatment of pancreatic cancer and should be considered for future human clinical trials.

## 1. Introduction

Sodium selenite has been found to have chemoprotective and antitumor capacities at non-toxic doses [1,2]. Some studies have shown that a low dose of selenite in supplementation with chemotherapy could help to reduce its side effects [1,3,4]. In addition, selenite has been found to possess a potent antitumor capacity and selectivity for tumor cells. This antitumor capacity has been demonstrated in vitro in lymphoma, leukemia, gastric, mesothelioma and colorectal carcinoma cell lines [5,6,7,8,9,10]. In vivo studies have also been conducted in mice with ovarian, prostate and colon tumor inocula, with satisfactory results [11,12,13]. By contrast, not enough clinical trials have been developed to evaluate improvements in the prognosis of cancer patients [14].

Sodium selenite is an inorganic oxo-salt used as a source of selenium, which is present in selenoproteins, such as glutathione peroxidase and thioredoxin reductase [15]. These proteins are involved in the elimination of reactive oxygen species (ROS), which are free radicals that induce damage by oxidative stress to proteins and DNA. However, at high doses, selenite becomes toxic to cells, including tumor cells. Selenite is an extremely reactive anion that is rapidly metabolized by the cell using a large amount of reducing power in the form of both reduced glutathione (GSH) and reduced thioredoxin (TRX), which perform vital functions at the cellular level, the most important being the detoxification of the ROS. Therefore, selenite-mediated depletion of GSH and TRX leaves the cell defenseless against ROS, resulting in cell death due to the severe DNA, protein and/or ribonucleotide damage [16]. In fact, it has been shown that ROS may generate cellular damage by (i) a mismatch of nitrogenous bases that activates the MMR (mismatch repair) system leading to futile repair cycles and apoptosis whether or not dependent on p53 (i.e., p73) [17,18]; (ii) double-strand breaks that, if not repaired, activate apoptosis via p53; and (iii) oxidative stress capable of inducing mitochondrial damage causing release of cytochrome c, activation of apoptosis (mitochondrial pathway) and autophagy. In addition, selenite-mediated TRX depletion, which causes ASK1 factor release, activates p38/JNK-dependent cell death [18], while selenite-mediated GSH reduction, which inactivates certain intracellular toxic agents, prevents the cell from defending itself from certain toxins (e.g., chemotherapeutic drugs) [16]. According to some authors [8], selenite could also directly block the active center of glutathione reductase and thioredoxin reductase, which are responsible for regenerating GSH and TRX, thereby decreasing the tumor cell’s ability to avoid the effect of drugs. Interestingly, in vitro and in vivo studies showed that tumor cells are more sensitive to selenite than normal cells. Paradoxically, cells highly resistant to traditional chemotherapy within tumors have been found to be the most sensitive to selenite exposure. Olm et al. [19] explained this phenomenon by MRP channel-based resistance mechanisms. The presence of these proteins in tumor cells allows chemotherapeutic agents to be expelled together with GSH (separately or conjugated). In these tumor cells, selenite—previously reduced to HSe^-^—is incorporated to a greater extent. Therefore, chemoresistant tumor cells (which expel more reducing power, in this case, GSH) would better incorporate the selenite and would suffer more damage [19]. Other selenium compounds have also demonstrated redox-based antitumor activity, such as methylselenic acid [20], selenomethionine [21] and sodium selenate [22]. In fact, Qiu et al. [20] used methylselenic acid against breast cancer in vitro and in vivo with positive results demonstrating significant inhibition of cancer cell growth by blocking JAK2 and STAT3. According to Goel et al. [21], selenomethionine induced apoptosis via the p53 pathway in HCT116 and RKO colon cancer cells. Choi et al. [22] used KBV20C oral cancer cells to demonstrate the chemosensitizing effect of sodium selenate with the chemotherapeutic agent vincristine. These authors also demonstrated that this effect was independent of *p*-glycoprotein inhibition.

On the other hand, pancreatic ductal adenocarcinoma, a malignant tumor from cells of the exocrine pancreas, is an uncommon tumor (2.1% of all tumors) mostly diagnosed between 65 and 75 years of age and with an increasing incidence, especially in the last few years [23,24]. It is characterized by a delayed diagnosis and a very poor prognosis. In fact, most of these tumors (70%) are diagnosed in stage III–IV, when they are already inoperable and have produced locoregional and/or hepatic metastases. In these cases, the only treatment available is chemotherapy. The most commonly used chemotherapeutic agents are antimetabolites, such as gemcitabine (GMZ) and 5-fluorouracil (5-FU); microtubule-depolymerizing agents, such as albumin bound paclitaxel (Abraxane); intercalating agents, such as oxaliplatin (OXA); or topoisomerase inhibitors, such as irinotecan (IRI). Despite the aggressiveness of these treatments, the survival of patients with pancreatic cancer barely reaches one year. One of the main causes of treatment failure is that pancreatic cancer cells show high drug resistance mediated by MDR (multiple drug resistance) proteins, such as *p*-glycoprotein or MRP, which expel drugs from the cell [25]. The combination of chemotherapeutic agents, such as GMZ–Abraxane (IMPACT clinical trial) or FOLFIRINOX (PRODIGE clinical trial), and the use of PAPR inhibitors have been tested to improve patient response. In the latter case, Olaparib increased survival in patients with BRCA-mutated pancreatic cancer who previously responded to FOLFIRINOX or FOLFOX (POLO clinical trial) [26].

In this context, the aim of this article is to study the antitumor capacity of sodium selenite alone and in combination with chemotherapy (GMZ), focusing on pancreatic cancer and the molecular mechanisms involved in its antitumor activity. Our results show that sodium selenite improves the response of pancreatic cancer to GMZ, supporting its potential use as a new strategy in the treatment of this type of tumor.

## 2. Materials and Methods

### 2.1. Cell Culture, Sodium Selenite and Drug Treatments

The human pancreatic cancer cell line PANC-1 was purchased from the Center of Scientific Instrumentation (CIC-Granada University), and the mouse pancreatic cancer cell line Pan02 was kindly provided by Dr. Lars Ivo Partecke, University of Greifswald, Germany. Both cell lines were cultured using DMEM medium supplemented with 10% fetal bovine serum (FBS) and 1% streptomycin and amphotericin B mixture (ATB). Cells were maintained in an incubator at 37 °C and 5% CO_2_ humidified atmosphere and passaged using 0.25% trypsin and 0.02% EDTA. Sodium selenite was dissolved in DMEM at the stock concentration of 578.03 mM. GMZ was obtained from Sigma (Sigma-Aldrich, Saint Louis, MO, USA) and dissolved in distilled water at the stock concentration of 379.94 mM. Benzamide was dissolved in DMEM at the 1981.18 µM stock concentration and was obtained from Sigma (Sigma-Aldrich, Saint Louis, MO, USA).

### 2.2. Cell Viability Analysis

Cells were seeded at densities of 5 × 10^3^ cells/well in PANC-1 and 4 × 10^3^ cells/well in Pan02 in 24-well plates with culture medium (400 µL), incubated overnight, and exposed to sodium selenite (from 1 to 30 µM), GMZ (from 0.025 to 5 µM) and both sodium selenite and GMZ (different concentrations) for 72 h. Then, cells were fixed with trichloroacetic acid (TCA) at 10% for 20 min at 4 °C. Once dried, the plates were stained with 0.4% sulforhodamine B (SRB) in 1% acetic acid (20 min, in agitation). After three washes with 1% acetic acid, SRB was solubilized with Trizma^®^ (10 mM, pH 10.5). Finally, the optical density (OD) of the dye was measured with a Titertek Multiskan colorimeter (Flow, Irvine, CA, USA) at 492 nm. The percentages of proliferation (Pf%) and cytotoxicity (Ct%) were calculated with Equation (1) Pf% = (sample OD/negative control OD) × 100 and Equation (2) Ct% = 100 − Pf%. In addition, IC50 (GraphPad Prism 6 Software, La Jolla, CA, USA) was determined according to Equation (3) Cell survival (%) = ((Treated cells OD − blanck)/(Control OD − blanck) × 100. In order to calculate synergy values in treatment combinations, the combination index equation (CIE), which defines synergism (CI < 1), additive effect (CI = 1) and antagonism (CI > 1), was obtained using CompuSyn software [27].

### 2.3. Cell Cycle Study

Cells were seeded in 6-well plates at a density of 15 × 10^4^ cells/well in PANC-1 and 10 × 10^4^ cells/well in Pan02 in 1.5 mL of complete DMEM. After 24 h, the culture medium was removed and a serum-free culture medium was added to arrest the cell cycle. Then, culture cells were exposed to different treatments (48 h), trypsinized, centrifuged (1500 rpm, 3 min) and fixed in 70% ethanol (4 °C, 60 min). After removing ethanol by centrifugation (1500 rpm, 3 min), the pellets were processed using the PI/RNASE solution kit (Inmunostep, Salamanca, Spain). Samples were analyzed in a BD FACSCanto II flow cytometer (Becton Dickinson, San Jose, CA, USA) using FlowJo software.

### 2.4. Mitochondrial Membrane Potential Analysis

The 1,1′,3,3,3′,3′-hexamethylindodicarbo-cyanine (DiIC1), a fluorescent dye that accumulates in mitochondria with depolarizing membrane potentials, was used to determine the modulation of mitochondrial potential in apoptotic-like cells. Cells were seeded in 6-well plates at a density of 10 × 10^4^ cells/well, exposed to different treatments (48 h), trypsinized and washed in PBS and centrifuged (2500 rpm, 3 min). Then, the pellets were resuspended in DMEM (without FBS and ATB) and incubated with DiIC1 (10 µM) and PI (1 mg/mL) for 20 min in darkness (ImmunoStep, Salamanca, Spain). Finally, cells were centrifuged (1500 rpm, 3 min), resuspended in PBS and analyzed with a flow cytometer BD FACSCanto II (Becton Dickinson, San Jose, CA, USA) using FlowJo software.

### 2.5. Benzamide Treatment Assays

Cells were seeded in 24-well plates at a density of 15 × 10^3^ cells/well in PANC-1 and 4 × 10^3^ cells/well in Pan02 and treated with the poly-ADP-ribose-polymerase (PARP) inhibitor benzamide (from 10 to 30 µM) (Sigma-Aldrich, Saint Louis, MO, USA) and with benzamide in association with selenite (different concentrations) for 72 h. Then, a sulforhodamine B proliferation assay was carried out as described above.

### 2.6. Apoptosis-Inducing Factor Immunofluorescence Assay

An immunofluorescence assay was used to determine apoptosis-inducing factor (AIF) expression in culture cells after 24 h of different treatments. Cells were included in 4% formaldehyde in PBS at room temperature (RT) (10 min), washed with PBS (two times) and neutralized (for residual formaldehyde) using 50 mM NH4Cl in PBS (10 min, RT). After one wash with PBS (5 min, 4 °C), cells were permeabilized with 0.5% Triton X-100 in PBS (5 min, 4 °C). Cell preparations were incubated with 1% bovine serum albumin in PBS (10 min, RT) and exposed to the primary anti-AIF antibody (Cell Signaling Technologies, Barcelona, Spain) (1/200 dilution, 1 h, RT). After PBS washing (three times), cell preparations were incubated with a secondary antibody conjugated to Alexa-488^®^ (Cell Signaling Technologies, Barcelona, Spain) (1/500 dilution, 30 min, RT). 4,6-diamidino-2-phenylindole (DAPI) (1/1000 dilution) was used to stain nuclei. The cells were observed by fluorescence microscopy (Leica Microsystems, Wetzlar, Germany).

### 2.7. Western-Blot Assay of Phosphorylated p38

Cells exposed to different treatments (72 h) (see above) were collected and centrifuged, and total proteins were extracted with RIPA lysis buffer (Sigma-Aldrich, Saint Louis, MO, USA) to determine protein concentration using Bradford. For electrophoresis, 40 µg of proteins from each sample were heated at 95 °C for 5 min and separated in 10% SDS-PAGE gel. Fractions were transferred to a nitrocellulose membrane (45 µm pore size) (Millipore, Burlington, MA, USA), blocked in 5% milk in PBS supplemented with 0.1% Tween-20 (Bio-Rad, Hercules, CA, USA) for 1 h and incubated with the anti-p38 rabbit primary antibody (1:1000) (Cell Signaling Technologies, Barcelona, Spain) overnight at 4 °C. Then, a secondary antibody (Sigma-Aldrich, Saint Louis, MO, USA) was added (1:5000). The membranes were revealed by chemiluminescence (Amersham Biosciences, Saint Louis, MO, USA). β-actin detection (Sigma-Aldrich, Saint Louis, MO, USA) served as an internal control. The gels were analyzed using Quantity One analytical software (Bio-Rad, Hercules, CA, USA).

### 2.8. Wound Healing Assay

To determine the tumor cell migration capacity of cell lines and, thus, their invasiveness, an in vitro migration assay was performed. Pancreatic cancer cells were seeded in 6-well plates and grown to 90% confluence in standard culture conditions. Then, they were treated with sodium selenite or GMZ (1 and 3 µM of selenite (IC10 and IC25) and 0.05 µM of GMZ (IC10)) for 72 h. A “wound” was manually performed with a sterile tip following Grada et al. [28], and the medium was replaced with serum-free DMEM. Images were obtained at different times (24, 48 and 72 h) to evaluate the percentage of migration by measuring the area free of tumor cells (Image J software v.1.53i, NIH, Besthesda, MD, USA) (https://imagej.nih.gov/, accessed on 14 October 2020).

### 2.9. Multicellular Tumor Spheroid Assays

Multicellular tumor spheroids (MTS) from PANC-1 and Pan02 were generated using our previously described protocol [29]. Briefly, PANC-1 and Pan02 cells (15 × 10^3^ and 25 × 10^3^ cells/well, respectively) were seeded in a 96-well plate coated with 1% (*w*/*v*) agarose and with 150 μL of complete DMEM. For PANC-1 MTS formation, the protocol was slightly modified [30] by supplementing DMEM with methylcellulose (0.24%). PANC-1 and Pan02 MTS were treated with sodium selenite, GMZ and combined treatments for two cycles of 72 h. Untreated MTS were used as controls. In addition, cell growth inhibition in MTS was also studied using CCK8 (Cell Counting Kit 8) (AbCam, Cambridge, UK).

### 2.10. Colony Formation Assay

PANC-1 and Pan02 cells were treated with sodium selenite, GMZ and combined treatments. After 72 h, cells were seeded in 12-well plates (600 cell/well) with 1 mL of DMEM. After three and six days for Pan02 and PANC-1 colony formation, respectively, the wells were washed twice using 1 mL of PBS and were fixed using 1 mL/well cold 70% methanol (RT, 30 min). Then, cells were washed twice with water and the plates were dried overnight. Colonies were stained using 1 mL/well 0.5% crystal violet in 70% methanol (15 min) on an orbital shaker. After that, the plates were washed three times using water and they were dried overnight. Finally, the number of colonies was counted.

### 2.11. CSC Spheroids Formation

PANC-1 cells were incubated with different treatments (sodium selenite, GMZ and combined treatment) for 72 h. Then, the remaining cells were collected and 3000 cells were seeded in 24-well plates coated with 1% (*w*/*v*) agarose and with 300 μL of conditioned medium (composed of HAM F12, 1% streptomycin-penicillin, hydrocortisone 1 µg/mL, heparin 4 ng/mL, insulin–transferrin–selenite—ITS—10 µg/mL, B27 1×, EGF 10 µg/mL, FGF 20 µg/mL) for pancreatic cancer stem cells (CSCs). After 10 days, once the spheroids were formed, they were counted in the different treatment groups.

### 2.12. In Vivo Tumor Growth Analysis

Brown and albino female C57BL/6 mice (weight 18–20 g, age 6 weeks) (Charles River Laboratories Inc, Wilmington, MA, USA) were housed in colony cages with free access to water and food prior to the experiments and with controlled light and temperature (22 ± 2 °C, and 12 h light–dark cycle). All animal studies were approved by the Ethics Committee on Animal Experimentation of the University of Granada (03-09-2017-118) and in accordance with international standards (European Communities Council Directive 2010/63). Subcutaneous tumors were induced with the Pan02 cell line by injecting 5 × 10^5^ cells in the right hind flank of mice in a total volume of 100 µL of saline serum. When tumors were palpable, the mice were randomly divided into four groups (*n* = 13 black, n = 5 albine for each group in both cases) and were treated with selenite (3 mg/kg), GMZ (25 mg/kg) or selenite + GMZ (association therapy). Each group was treated every three days for a total of 10 doses (intraperitoneal administration). In addition, one group was treated with saline solution (control, *n* = 13). The following formula was used to calculate the tumor volume: V (mm^3^) = (a × b^2^ × π)/6, where “a” is the largest diameter of the tumor, and “b” is the largest diameter perpendicular to “a”. Tumor growth values were measured in mm^3^/day. In addition, mice survival was measured. After the experiments were completed, the mice were sacrificed with pentobarbital, and the tumors were excised.

### 2.13. Bioimaging

An IVI Spectrum (PerkinElmer, Waltham, MA, USA) study using glucose labeled with a fluorophore (RediJect 2-DeoxyGlucosone (DG)-750, PerkinElmer, Waltham, MA, USA) was carried out in albino mice groups after 5 and 10 treatment cycles to assess the viability of the tumor tissue, and the mice were analyzed after 3 h of administration.

### 2.14. Histological Analysis

Resected tumors were fixed in formaldehyde, included in paraffin and cut with a rotation microtome (Leica, Wetzlar, Germany). After deparaffinization and hydration, the sections were stained with hematoxylin–eosin and the pentachrome method [31]. Immunofluorescence was used to determine Ki67 and MMP9 expression (BD 550,609 and ab38898 at 1:50 and 1:200, respectively) (BD Biosciences, San Jose, CA, USA; AbCam, Cambridge, UK). Alexa-488^®^ and 647^®^ (Cell Signaling Technologies, Barcelona, Spain) were used as secondary antibodies (1:200 for both). The images were obtained with a photographic microscope (Leica, Wetzlar, Germany).

### 2.15. Statistical Analyses

All the results were expressed as mean ± standard deviation (SD) of three or more replicates. Statistical analysis was performed using Student’s t-tests and one-way ANOVA with post hoc Tukey tests (SPSS v.15, SPSS, Chicago, IL, USA). Mice survival was evaluated with the Kaplan–Meier method. Finally, the log-rank test was used to compare the proportion of living mice between groups. All tests were performed with the Statistical Package for the Social Sciences (SPSS) v. 15.0 software, and differences were considered statistically significant at a *p*-value < 0.05.

## 3. Results

### 3.1. Sodium Selenite Reduces Cell Viability in Pancreatic Cancer Cells

**Figure 1:**
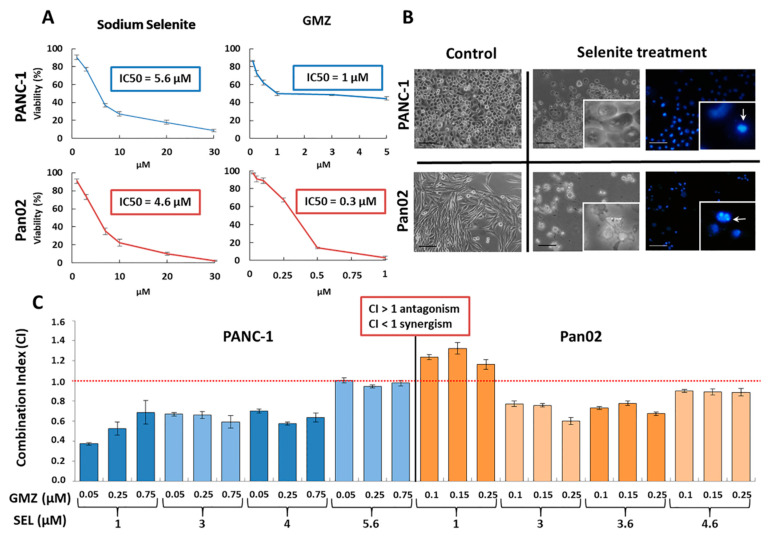
Effect of sodium selenite on pancreatic cancer cells. (**A**) Cell viability graphs and IC50 values of sodium selenite and GMZ in PANC-1 and Pan02 cells. Cells were exposed to sodium selenite and GMZ at different doses for 72 h. (**B**) Morphological changes in PANC-1 and Pan02 cells after exposure to sodium selenite (magnification 10×; detail magnification 15×). Hoestch staining showed nuclear fragmentations (arrows) indicating the presence of apoptotic bodies (apoptotic-like cell death). (**C**) Combination index (CI) of sodium selenite and GMZ in PANC-1 and Pan02 cells (CI > 1, antagonism; CI < 1, synergism). GMZ (gemcitabine); SEL (sodium selenite).

Sodium selenite significantly inhibited pancreatic cancer cell viability in a time- and dose-dependent manner, showing IC50 values of 5.6 and 4.6 µM for PANC-1 and Pan02 cell lines, respectively (Figure 1A). The decrease in cell density after 72 h of selenite exposure was accompanied by morphological changes, including a clear loss of the flat, polygonal shape of cells, detachment from the culture plate and apoptosis-like signals in the nucleus (Hoestch stained images) in both PANC-1 and Pan02 cells (Figure 1B). Interestingly, the combination of selenite (IC10, IC25, IC40 in PANC-1 and IC25, IC40, IC50 in Pan02) and GMZ (IC10, IC25 and IC40 in both cell lines) revealed a synergic effect on cell death in both PANC-1 and Pan02 cells (CI < 1). Some loss of effect was observed at IC10 of selenite in the Pan02 cell line and at IC50 of selenite in the PANC-1 cell line (Figure 1C).

### 3.2. Modulation of Cell Cycle and Mitochondrial Membrane Potential by Sodium Selenite

**Figure 2:**
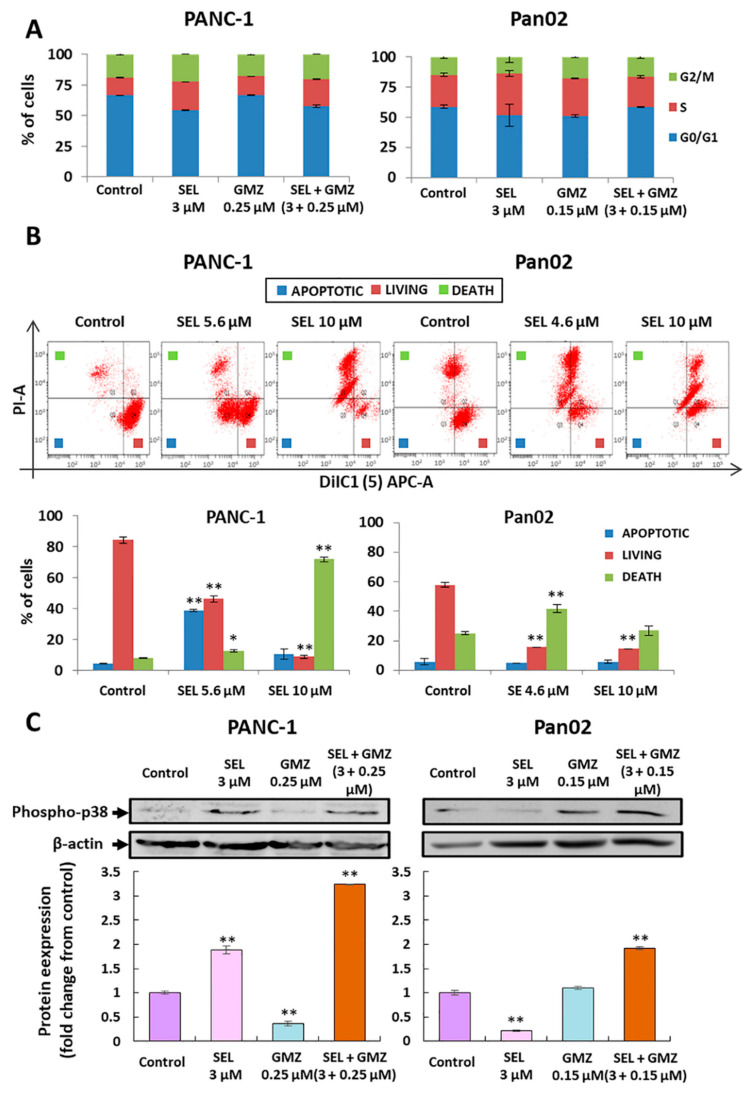
Cell cycle, mitochondrial membrane potential and molecular changes in pancreatic cancer cells treated with sodium selenite. (**A**) Modulation of cell cycle by sodium selenite. Sodium selenite was used alone and in association with GMZ in PANC-1 and Pan02 pancreatic cancer cells. (**B**) Apoptotic-like changes (PI-A) and mitochondrial depolarization (DiIC1 (5)) in PANC-1 and Pan02 pancreatic cancer cells after selenite treatment (upper graph). Percentage of living, apoptotic-like and dead cells based on the previous data (lower graph). (**C**) Representative Western blot and densitometric analysis of the phospho-p38 protein expression in pancreatic cancer cells. PANC-1 and Pan02 cells were treated with sodium selenite, GMZ and sodium selenite + GMZ. Beta-actin was used as control. All data are presented as mean ± standard deviation of three independent experiments; * *p* < 0.05 and ** *p* < 0.01 vs. respective control group. GMZ (gemcitabine); SEL (sodium selenite).

PANC-1 and Pan02 pancreatic cells treated with sodium selenite, GMZ or sodium selenite + GMZ did not show substantial changes in cell cycle (65% G0/G1 phase, 15% phase S, 20% G2/M phase) (Figure 2A). On the other hand, membrane potential assays using DiIC1 revealed that sodium selenite induced mitochondrial depolarization. As shown in Figure 2, these changes were more evident in PANC-1 cells, where 40% of cells with mitochondrial depolarization and low PI (apoptosis-like cell death) were observed after exposure to 5.6 µM selenite (Figure 2B).

### 3.3. Sodium Selenite and Its Association with Gemcitabine: Molecular Analysis

In order to determine the molecular mechanisms involved in the enhanced effect between selenite and GMZ, we analyzed the phospho-p38, a 38 kDa protein that activates apoptosis by a p53 independent way. As shown in Figure 2C, a significant increase (twofold) in the expression of phospho-p38 in PANC-1 cells treated with IC25 of sodium selenite compared to those treated with IC25 of GMZ was detected. However, the expression of phospho-p38 in Pan02 cells treated with IC25 of selenite showed lower levels than the control and GMZ-treated cells, with the latter two showing similar levels of phospho-p38. Interestingly, both PANC-1 and Pan02 cells treated with the sodium selenite + GMZ association (IC25 of both compounds) showed an increased expression of phospho-p38 in comparison with selenite or GMZ alone (threefold and twofold, respectively).

**Figure 3:**
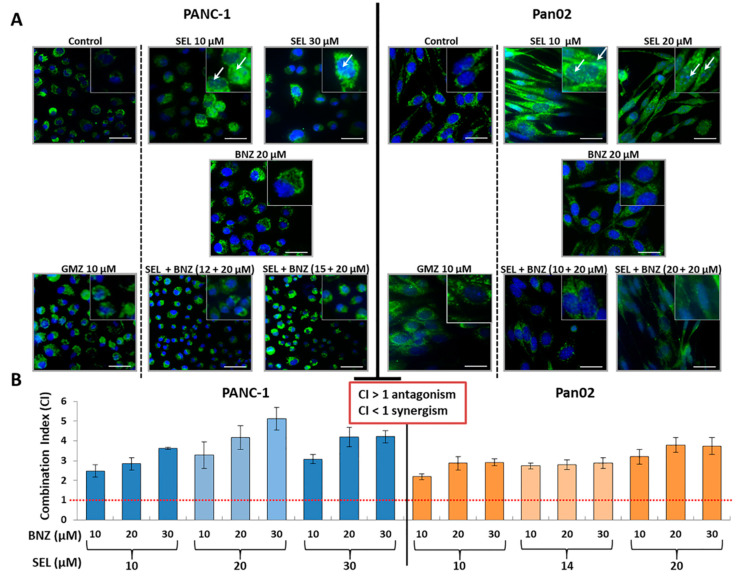
Expression of AIF: immunofluorescence analyses and effect of benzamide. (**A**) Immunofluorescence analyses of AIF expression in PANC-1 and Pan02 pancreatic cells treated with selenite, GMZ, BZD and selenite + BZD at different concentrations. Representative images of mitochondrial staining and nuclear translocation depending on the treatment analyzed. Square × 2 magnification of the principal image. (**B**) Combination index (CI) of PARP inhibition studies using BZD and selenite + BZD. GMZ (gemcitabine); SEL (sodium selenite); BNZ (benzamide).

On the other hand, the expression of AIF, a nuclear effector protein of parthanatos, was analyzed in relation to the mechanism of action of selenite. An immunofluorescence study showed that sodium selenite induced a large AIF nuclear location in both PANC-1 and Pan02 cells (Figure 3A). By contrast, GMZ treatment did not induce nuclear location of the AIF protein. In addition, to corroborate the effect of selenite on the expression of AIF, we used BZD, a known PARP inhibitor (see Methods). In both cell lines, BZD seemed to inhibit or decrease the selenite-mediated nuclear translocation of AIF. As shown in Figure 3B, BZD concentrations lower than IC10 inhibited sodium selenite cytotoxicity in both PANC-1 and Pan02 cell lines at all the combination doses studied (CI > 1). In addition, BZD inhibited or decreased the selenite-mediated nuclear translocation of AIF (Figure 3B).

### 3.4. Sodium Selenite Inhibited Cell Colony Formation and Migration Capacity in Pancreatic Cancer Cells

**Figure 4:**
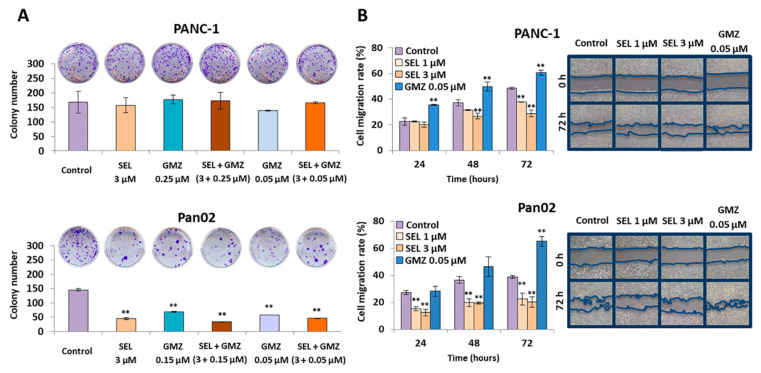
Effect of sodium selenite on colony formation and cell migration capacity. (**A**) Colony growth analysis of PANC-1 and Pan02 after selenite, GMZ and both selenite + GMZ treatment (72 h). (**B**) Cell migration analysis. Representative optical microscopy images and graphical representation of the modulation of migration capacity of PANC-1 and Panc02 cells after exposure to sodium selenite and GMZ at different times (0 and 72 h). All data are presented as mean ± standard deviation of three independent experiments; ** *p* < 0.01 vs. respective control group. GMZ (gemcitabine); SEL (sodium selenite).

As shown in Figure 4A, sodium selenite significantly reduced the formation of colonies in all treatment groups. This effect was visible in the Pan02 cell line, in which up to 60–70% reduction of colony formation was demonstrated with the use of 3 µM selenite, 0.05 and 0.15 µM GMZ (approximate IC10 and IC25 values) and the combination of both. In PANC-1, results were not significant. On the other hand, exposure to sodium selenite significantly decreased migration capacity in both Pan02 and PANC-1 pancreatic cancer cells. In fact, sodium selenite at 1 and 3 µM (approximate IC10 and IC25 values) doses reduced Pan02 cell migration (16.2 and 18.5%, respectively) after 72 h of exposure. In PANC-1 cells, a 10.6 and 19.6% migration decrease was detected with the use of selenite at 1 and 3 µM doses, respectively, at the same times (Figure 4B).

### 3.5. Sodium Selenite Inhibited Growth of Multicellular Tumor Spheroids from Pancreatic Cancer Cells

**Figure 5:**
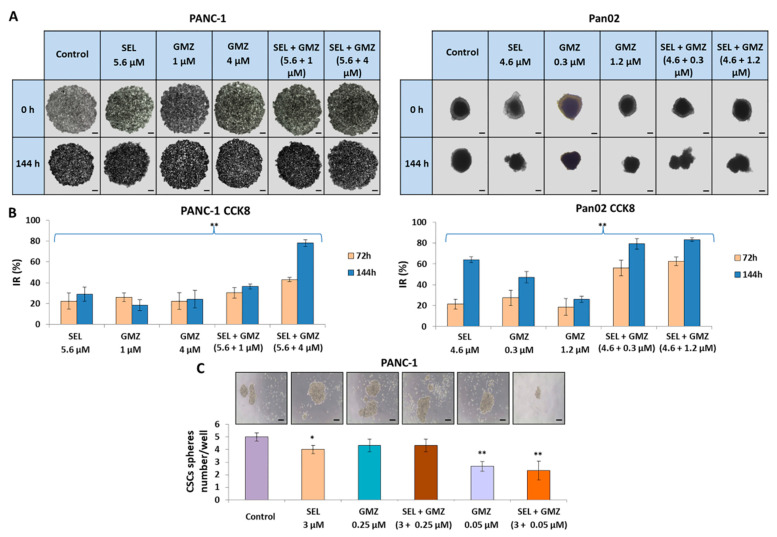
Cell growth inhibition of MTS from PANC-1 and Pan02 pancreatic cancer cell lines after selenite treatment. (**A**) PANC-1 and Pan02 MTS in culture with different treatments at 0 and 144 h. (**B**) CCK8 analysis on the PANC-1 and Pan02 MTS at 72 and 144 h. Data are presented as mean ± SD of octuplicate cultures. ** *p* < 0.01 vs. respective control group. (**C**) Study of CSC sphere formation. Data are presented as mean ± SD of three independent experiments; * *p* < 0.05 and ** *p* < 0.01 vs. respective control group. GMZ (gemcitabine); SEL (sodium selenite).

Multicellular tumor spheroids (MTS) from PANC-1 and Pan02, an experimental model that mimics tumors in vivo, were used to determine the penetrability and antitumor effect of sodium selenite alone and in combination with GMZ. As shown in Figure 5A,B, both PANC-1 and Pan02 MTS showed a significant reduction in tumor viability (CCK8 assay) after exposure to selenite alone and selenite + GMZ. Specifically, PANC-1 MTS treated with selenite (5.6 µM, IC50) and GMZ (1 µM and 4 µM, IC50 and IC50 × 4) showed 28.9, 18.5 and 24.3% cell growth inhibition, respectively, at 144 h. Interestingly, the combination of both drugs inhibited cell growth by 36.4% (5.6 µM selenite + 1 µM GMZ) and 78.03% (5.6 µM selenite + 4 µM GMZ) at 144 h. In the case of Pan02 MTS, selenite (4.6 µM, IC50) and GMZ (0.3 µM and 1.2 µM, IC50 and IC50 × 4 showed 64.02, 47.14 and 25.7% cell growth inhibition, respectively, at 144 h. Finally, the combination of both drugs inhibited 79.1% (4.6 µM selenite + 0.3 µM GMZ) and 83.2% (4.6 µM selenite + 1.2 µM GMZ) at 144.

### 3.6. Sodium Selenite Modulated Cancer Stem Cells Sphere Formation

In order to analyze how a pretreatment of the cells with selenite would modify the ability of CSCs to form spheres at 72 h, CSC spheres were induced with PANC-1 living cells. As shown in Figure 5C, sphere formation decreased when cells were previously treated with selenite and GMZ, as well as with the combined treatment. However, these differences were not pronounced, probably due to the lower number of spheres forming the baseline.

### 3.7. Sodium Selenite Inhibited Growth of Pancreatic Cancer Tumor and Enhanced Gemcitabine Effect: In Vivo Assays

**Figure 6:**
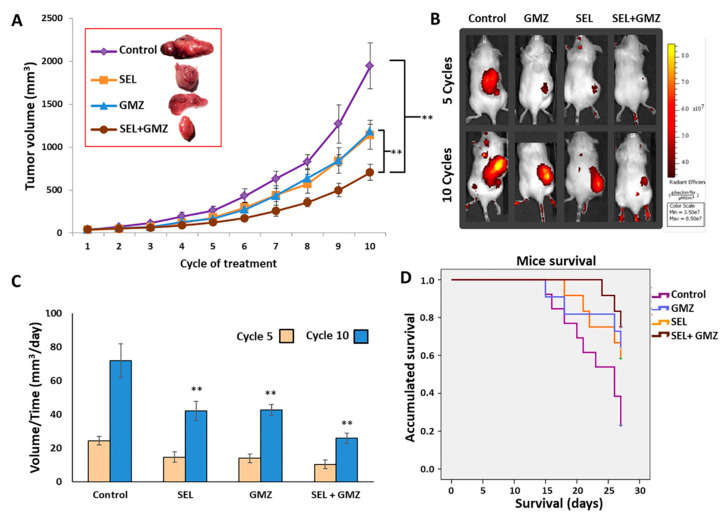
In vivo tumor growth inhibition and mice survival after treatment with sodium selenite. (**A**) Graphic representation of pancreatic tumor (Pan02 cancer cells) volume growth in C57BL/6 mice. Mice were treated with sodium selenite, GMZ and sodium selenite + GMZ. Untreated mice were used as controls. Data are presented as mean ± SD (*n* = 13). ** Significant inhibition of tumor growth comparing treatments and comparing treatments with control (*p* < 0.01). (**B**) IVI Spectrum analysis of mice after different treatment protocols (5 and 10 cycles). Images were obtained after 3 h of (DG)-750 injection. (**C**) Tumor growth inhibition in volume/time (mm^3^/day) (** *p* < 0.01, comparing treatments and comparing treatments with control). (**D**) Kaplan–Meier curves of mice bearing subcutaneous tumors. Data were analyzed according to mice survival in each group. Comparison between treatment groups was performed using the log-rank test. GMZ (gemcitabine); SEL (sodium selenite).

Immunocompetent C57BL/6 and C57BL/6 albino mice bearing a Pan02 tumor were used to determine the antitumor potential of sodium selenite in vivo. First, as shown in Figure 6, a significant reduction in tumor volume of C57BL/6 mice treated with sodium selenite + GMZ was detected (*p* < 0.01) in comparison with the untreated (control) group. At the end of the study (day 27, 10 cycles of treatment), tumor growth was inhibited by up to 65% in mice treated with selenite + GMZ compared to the control group. Interestingly, the combined therapy (selenite + GMZ) also induced a greater tumor volume reduction compared to sodium selenite or GMZ used as monotherapy (up to 40% reduction). In the case of volume/time values, a significant reduction was observed in selenite + GMZ group with respect to the rest of the treatments and in selenite and GMZ in monotherapy relative to the control group. In the combination group, the reduction in tumor growth by volume/time was up to 50% relative to the control. Both selenite + GMZ and the sodium selenite treatment allowed a greater survival in comparison with the control group. In addition, the Kaplan–Meier curve showed a fourfold increase in the fraction of surviving mice when treated with selenite + GMZ in comparison with mice treated with GMZ alone and an eightfold increase with respect to the control mice (*p* < 0.05). Moreover, a complementary study in C57BL/6 albino mice bearing Pan02 cancer was carried out using the IVIS system. As shown in Figure 6B, although a partial response was detected in mice treated with both monotherapies (sodium selenite and GMZ) after 10 cycles, the greatest response was observed with the use of selenite + GMZ. Analysis of the removed tumors demonstrated a significantly smaller size in mice treated with the combined therapy (sodium selenite + GMZ) compared to monotherapy treatments (Figure 6).

### 3.8. Histological Analysis

**Figure 7:**
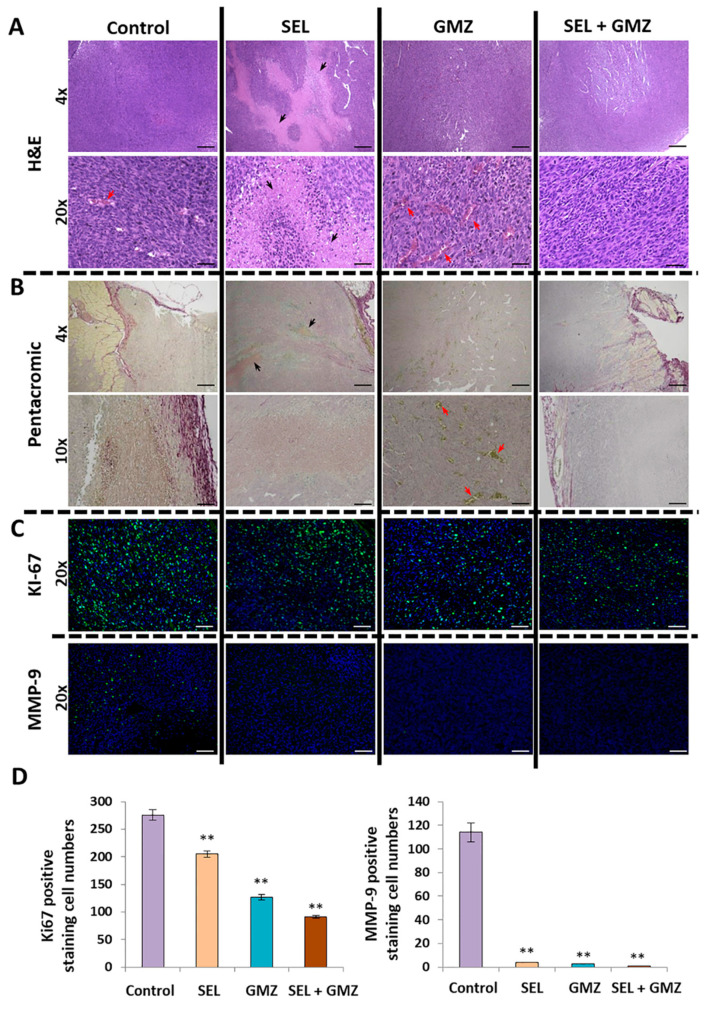
Histological analysis of resected tumors by histological basic staining and immunofluorescence. (**A**) Stained hematoxylin–eosin samples from the different treatment groups (4× and 20× magnification). (**B**) Samples stained with the new pentachromic method at 4× and 10×. This method highlights the visualization of blood vessels with red blood cells in yellow inside and thin layers of intimal collagen. (**C**) Immunofluorescence staining for Ki67 and MMP9 at 20×. (**D**) Graphical representation of immunofluorescence positive staining quantification by Image J. Data are presented as mean ± standard deviation of three independent experiments; ** *p* < 0.01 vs. respective control group. Scale bar 4× (200 µM), 10× (150 µM) and 20× (50 µM). GMZ (gemcitabine); SEL (sodium selenite).

Once the tumors were resected, basic histological stains (hematoxylin–eosin), histochemistry (new pentachrome method) and immunofluorescence for Ki67 and MMP9 were performed. Hematoxylin–eosin showed the predominance of wide areas of acidophilic cell necrosis in tumors treated with sodium selenite. By contrast, GMZ monotherapy induced a much larger number of blood vessels that were not detected with the use of selenite + GMZ treatment. Pentachromic staining supported these results, showing a significant increase in angiogenesis after GMZ in monotherapy but not after selenite + GMZ treatment. An in vitro study using conditioned media from selenite-treated Pan02 cells (1 and 10 µM) showed a significant decrease in the formation of vessels in HUVEC cells, indicating a clear reduction of their angiogenesis capacity (Supplementary Figure S1). On the other hand, a significant reduction in the expression of Ki67 was detected after the combined therapy in comparison to control (3.44-fold reduction), selenite and GMZ monotherapy (2.63- and 1.56-fold reduction, respectively), indicating a reduction in proliferation. In addition, the expression of MMP9 strongly decreased in all treatments assayed in comparison with control cells (Figure 7).

## 4. Discussion

Selenium, a crucial cofactor in endogenous antioxidative systems, has a controversial action in cancer. On the one hand, its deficiency could be associated with an increase in cancer incidence [2,30]. On the other hand, selenium may have a relevant role in cancer association therapy with conventional chemotherapeutic drugs since it has been shown to reduce their side effects or improve treatment efficacy [3,31]. In this context, we analyzed selenium for pancreatic cancer treatment, alone and in association with standard GMZ treatment.

In vitro studies demonstrated that sodium selenite has relevant antitumor activity in several types of tumors, including lymphoma, leukemia, gastric, mesothelioma and colorectal carcinoma [5,6,7,8,9,10], with an IC50 range between 1 and 20 µM. In a phase I clinical trial (SECAR clinical trial) with mainly lung cancer patients, similar plasma concentrations were tolerated, which were significantly lower than the maximum tolerated dose (MTD) [32]. The mechanism of action and the selectivity against tumors of selenite remain unclear but may be due to the intrinsic molecular mechanisms of the cells and the tumor microenvironment. In fact, Olm et al. [17] showed a process of selenite reduction (selenide, HSe^-^) in the acidic environment of the tumor that leads to sodium selenite activation, making it more permeable to the cell membrane. This phenomenon may be enhanced by drug resistant mechanisms (e.g., MRP), which decrease reduced glutathione within the tumor cell. Interestingly, pancreatic cancer cells showed elevated MRP expression [25]. In fact, the PANC-1 and Pan02 pancreatic cancer cell lines used in our study were characterized by high MRP expression [33,34]. The expression of MRP may favor selenite incorporation into and selectivity for tumor cells, which could explain the sensitivity of pancreatic cancer cells to selenite in both in vitro and in vivo experiments. In fact, both PANC-1 and Pan02 cell lines were highly sensitive to selenite treatment, as demonstrated by their IC50 value (5.6 and 4.6 µM, respectively).

**Figure 8:**
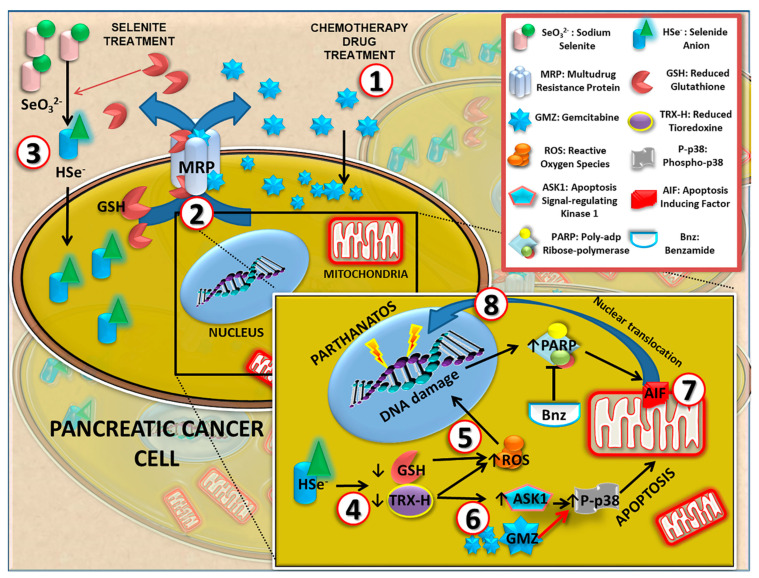
Molecular mechanisms involved in the anticancer activity of sodium selenite in pancreatic cancer cells. This model summarizes the mechanisms by which selenite can induce cell death in tumor cells and hypothesizes about the enhanced antitumor effect found when selenite and gemcitabine were associated. (1). Gemcitabine (GMZ) enters the cell cytoplasm. (2). GMZ is detoxified by MRP system conjugated with reduced glutathione (GSH). (3). Specifically, sodium selenite (SeO_3_ ^2-^) is reduced to selenide (HSe^-^) by GSH, which is expelled due to cellular detoxification processes via the MRP system. (4). Once inside the cell cytoplasm, selenite depletes GSH and reduced thioredoxin (TRX-H), leaving the cell defenseless against reactive oxygen species (ROS) and increasing them. (5). ROS cause DNA damage, which is repaired by poly-ADP-ribose polymerase (PARP), resulting in overexpression of this enzyme. Benzamide (Bnz) inhibits PARP as well as the subsequent processes of repair and cell death induced by this enzyme. (6). GMZ is a chemotherapeutic agent that induces apoptosis via phospho-p38 activation. As shown in the figure, SeO_3_ ^2-^ decreases TRX-H and releases the ASK1 factor, which phosphorylates p38. The synergy between GMZ and SeO_3_ ^2-^ could be related to the latter mechanism. (7) and (8). The accumulation of DNA damage due to ROS activates PARP to initiate the translocation of apoptosis-inducing factor (AIF) from the mitochondria to the cell nucleus, inducing cell death by parthanatos.

In addition, selenite was also able to enhance the activity of classic agents used in the treatment of pancreatic cancer, such as GMZ. A significant antitumor synergistic effect was observed when different cytotoxic doses of selenite and GMZ were associated in pancreatic adenocarcinoma PANC-1 and Pan02 cell lines. As demonstrated by cell cycle analysis and mitochondrial membrane potential analysis, sodium selenite caused an apoptosis-like-mediated cell death. However, previous studies such as that developed by Soukupová et al. [35] demonstrated that sodium selenite at cytotoxic doses diminished mitochondrial membrane potential but did not activate caspase 3 in a significant way. Parthanatos or cell death produced by PARP is activated when PARP has repaired extensive damage by base excision [36]. Selenite-mediated cytotoxicity, which causes reduced glutathione and thioredoxin depletion, leaves the cell defenseless to oxidative stress, which could activate this mechanism of cell death [8]. Our studies showed that PARP-mediated cell death could be involved in sodium selenite-induced apoptotic-like cell death. In fact, PARP inhibition by benzamide significantly reduced the antitumor effect of selenite. Moreover, a nuclear translocation of the AIF factor was detected in selenite-treated tumor cells. This mitochondrial factor is the effector of parthanatos and translocates to the nucleus when this cell death pathway is activated. Interestingly, the translocation was not detected in the combined treatment with benzamide, which supports the possible involvement of PARP in the mechanism of action of selenite. As shown in Figure 8, several molecular mechanisms may be involved in the antitumor action of sodium selenite in pancreatic cancer cells. Selenite causes a decrease in intracellular levels of reduced thioredoxin, which normally blocks the ASK1 factor. In turn, this factor is released and able to activate p38 (by phosphorylation), which induces cell death by apoptosis [37]. It has been demonstrated that p38 is the protein that mediates GMZ-induced apoptosis [38]. According to our results, the association of selenite and GMZ induced a higher p38 activation than both monotherapies, which could explain the synergy between cytotoxic doses of selenite and GMZ.

On the other hand, selenite induced a significant growth inhibition of pancreatic cancer cell-derived MTS, an in vitro system that mimics tumors in vivo. This effect may be related to the high diffusion capacity of selenite and the lower pH within the tumor spheroid, which would make it more susceptible to treatment [19]. Migration and colony formation assays indicated that selenite significantly reduced these parameters relative to the effect of GMZ (which enhanced migration of PANC-1 and Pan02 cells), suggesting a decrease in aggressiveness in this cell type. The paradoxical increase in tumor migration by GMZ has been described by Xu at al. [39] and is related to the activation of EGFR and STAT3 pathways by this drug. In addition, sodium selenite and GMZ showed an inhibitory effect against colony formation in Pan02 but not in PANC-1 cells. Moreover, the inhibition of tumor migration in Pan02 was also more evident. This phenomenon might be related to the fact that Pan02 cells show mesenchymal morphological features and SMAD4 mutations (alterations in the TGF-beta pathway), while PANC-1 cells display an epithelioid morphology and mutations in *KRAS* and *TP53* [40,41]. Furthermore, selenite demonstrated a decrease in blood vessel formation when an angiogenesis assay using the Pan02 cells was performed. These results were supported by the in vivo assay in which blood vessel development was assessed by histological methods (Figure 7). In addition, our assays on the ability of CSCs to form spheres in a conditioned medium after treatment showed that selenite affects this type of cell population more, reducing the number of spheres formed after previous treatment with selenite and with selenite + GMZ. The sensitivity of CSCs to selenite treatment could be related to the increased sensitivity of CSCs to oxidative stress as described by Liu and Wang [42]. In previous studies on PANC-1 CSCs, GMZ 0.05 showed greater inhibition than GMZ 0.25, a paradoxical finding that could be related to its high cytotoxicity against sensitive CSC populations and to the high replacement activity of CSCs resistant to GMZ 0.25. Further studies will be necessary in order to confirm this hypothesis.

Finally, to our knowledge, this is the first experimental in vivo study using selenite against pancreatic cancer, a chemoresistant tumor with poor prognosis and limited treatment options. In vivo assays with mice bearing pancreatic tumors clearly corroborated the antitumor activity of selenite, both alone and in combination with GMZ. Using doses and cycles of selenite and GMZ in a range commonly used in other studies with mice [43], we demonstrated that the combination of selenite–GMZ was more effective in terms of decreasing tumor growth, survival and metabolic response in comparison with the rest of treatments, including GMZ monotherapy. The in vivo antitumor potential of selenite associated with other antitumor agents (e.g., carmustine or cisplatin) has been demonstrated in prostate, ovary and colon tumors [11,12,13]. Caffrey et al. [9] showed that sodium selenite (1.5 mg/kg) in combination with cisplatin, but not alone, was effective in ovarian cancer. In addition, Thamilselvan et al. [11] showed that the association of selenite (1.5 mg/kg) and carmustine induced a higher antitumor effect against prostate cancer than both monotherapies, although no synergistic effect was detected. This study also revealed a significant antitumor activity of selenite against prostate cancer, but lower than that of carmustine. Our results using higher doses of selenite (3 mg/kg) associated with GMZ showed good tolerance, longer survival and significant tumor volume reduction (50%) compared to monotherapies (i.e., GMZ or selenite). Histological analysis verified that selenite and selenite + GMZ treatments decreased the expression of cell proliferation markers (i.e., Ki67) and that both treatments were able to inhibit MMP9, a protein closely related to the invasiveness of pancreatic cancer. Furthermore, selenite demonstrated a strong necrotic capacity and antiangiogenic activity, which are not observed with GMZ as monotherapy. In fact, Saeed et al. [44] already described the antiangiogenic capacity of selenite in colon tumors, which correlates with the decrease in the expression of proangiogenic factors such as VEGF in the analyzed samples.

## 5. Conclusions

In this article, we demonstrated for the first time the antitumor effect of sodium selenite against pancreatic cancer in cell cultures in vitro, three-dimensional models in vitro and in vivo assays. Furthermore, selenite not only showed an antitumor effect as monotherapy but also in combination with GMZ, showing an enhanced effect with GMZ. The mechanisms by which selenite acts on tumor cells (such as parthanatos or AIF-mediated cell death) were different from those of traditional chemotherapeutic agents, suggesting that selenite could be an adjuvant therapy to overcome the phenomena of resistance to traditional drugs. In addition, selenite demonstrated a significant effect against critical processes in tumor progression, such as colony formation, migration or the ability of CSCs to form spheres. In vivo assays demonstrated the efficacy of selenite (alone and in combination with GMZ) in increasing survival and in significantly reducing tumor volume and the expression of markers related to tumor aggressiveness and progression. Therefore, sodium selenite should be considered as a promising antitumor agent against pancreatic cancer, either alone or in combination with GMZ. However, future studies will be necessary to determine its potential clinical applicability and the plausibility of clinical trials.

## Data Availability

The data presented in this study are available in the article or Supplementary Files.

## Supplementary Materials

The following are available online at https://www.mdpi.com/article/10.3390/cancers13133169/s1, Figure S1: Effect of pancreatic cancer cell-conditioned media after sodium selenite treatment on angiogenesis.
