# Supplementary material for: The Antitumor Activity of Sodium Selenite Alone and in Combination with Gemcitabine in Pancreatic Cancer: An In Vitro and In Vivo Study
Source: Cancers (Basel). 2021 Jun 25;13(13):3169. doi: 10.3390/cancers13133169 (PMC8268835; doi:10.3390/cancers13133169)
Supplement: Supplementary file 1 [file cancers-13-03169-s001.zip › cancers-1241512-supplementary/Figure Original images.pdf]

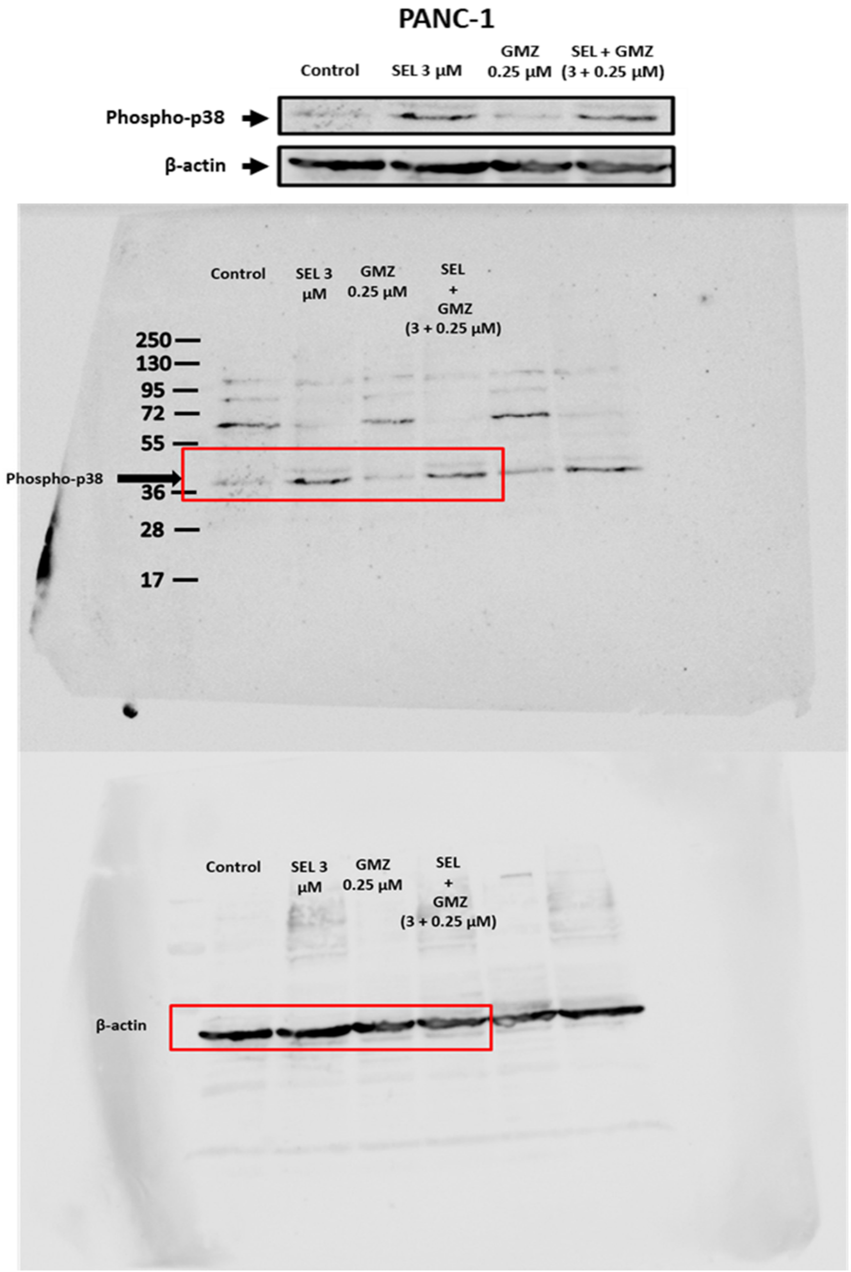
PANC-1
Control
SEL 3 µM
GMZ 0.25 µM
SEL + GMZ (3 + 0.25 µM)
Phospho-p38
β-actin
Control
SEL 3 µM
GMZ 0.25 µM
SEL + GMZ (3 + 0.25 µM)
250
130
95
72
55
36
28
17
Phospho-p38
Control
SEL 3 µM
GMZ 0.25 µM
SEL + GMZ (3 + 0.25 µM)
β-actin

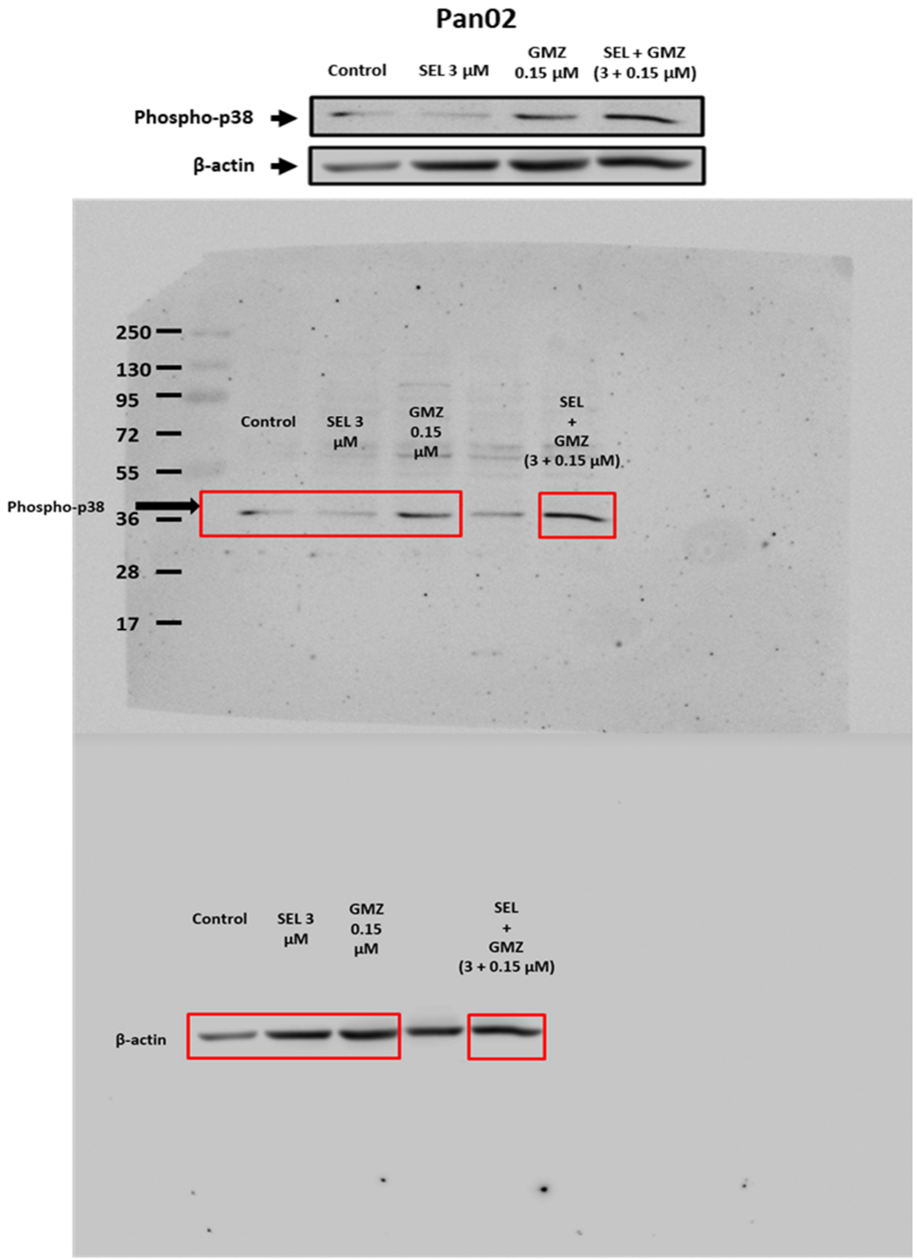
Pan02
Control
SEL 3 µM
GMZ 0.15 µM
SEL + GMZ (3 + 0.15 µM)
Phospho-p38
β-actin
250
130
95
72
55
36
28
17
Control
SEL 3 µM
GMZ 0.15 µM
SEL + GMZ (3 + 0.15 µM)
Phospho-p38
Control
SEL 3 µM
GMZ 0.15 µM
SEL + GMZ (3 + 0.15 µM)
β-actin

Original Western Blots of Figure 2C. Cells exposed to different treatments (72 h) were collected, centrifuged, and total proteins were extracted with RIPA lysis buffer (Sigma-Aldrich, Saint Louis, Missouri) to determine protein concentration using Bradford. For electrophoresis, 40 μg of proteins from each sample were heated at 95 °C for 5 min and separated in 10% SDS-PAGE gel. Fractions were transferred to a nitrocellulose membrane (45 μm pore size) *(Millipore),* blocked in 5% milk in PBS supplemented with 0.1% Tween-20 *(*Bio-Rad*)* for 1 hour and incubated with the anti-p38 rabbit primary antibody (1:1000) (Cell Signaling Technologies, Spain) overnight at 4 °C. Then, a secondary antibody (Sigma-Aldrich, Saint Louis, Missouri) was added (1:5000). The membranes were revealed by chemiluminescence (Amersham Biosciences, USA). β-actin detection *(*Sigma-Aldrich, Saint Louis, Missouri) served as an internal control.
