# Supplementary material for: The Antitumor Activity of Sodium Selenite Alone and in Combination with Gemcitabine in Pancreatic Cancer: An In Vitro and In Vivo Study
Source: Cancers (Basel). 2021 Jun 25;13(13):3169. doi: 10.3390/cancers13133169 (PMC8268835; doi:10.3390/cancers13133169)
Supplement: Supplementary file 1 [file cancers-13-03169-s001.zip › cancers-1241512-supplementary/Figure S1 . Suplem. material.pdf]

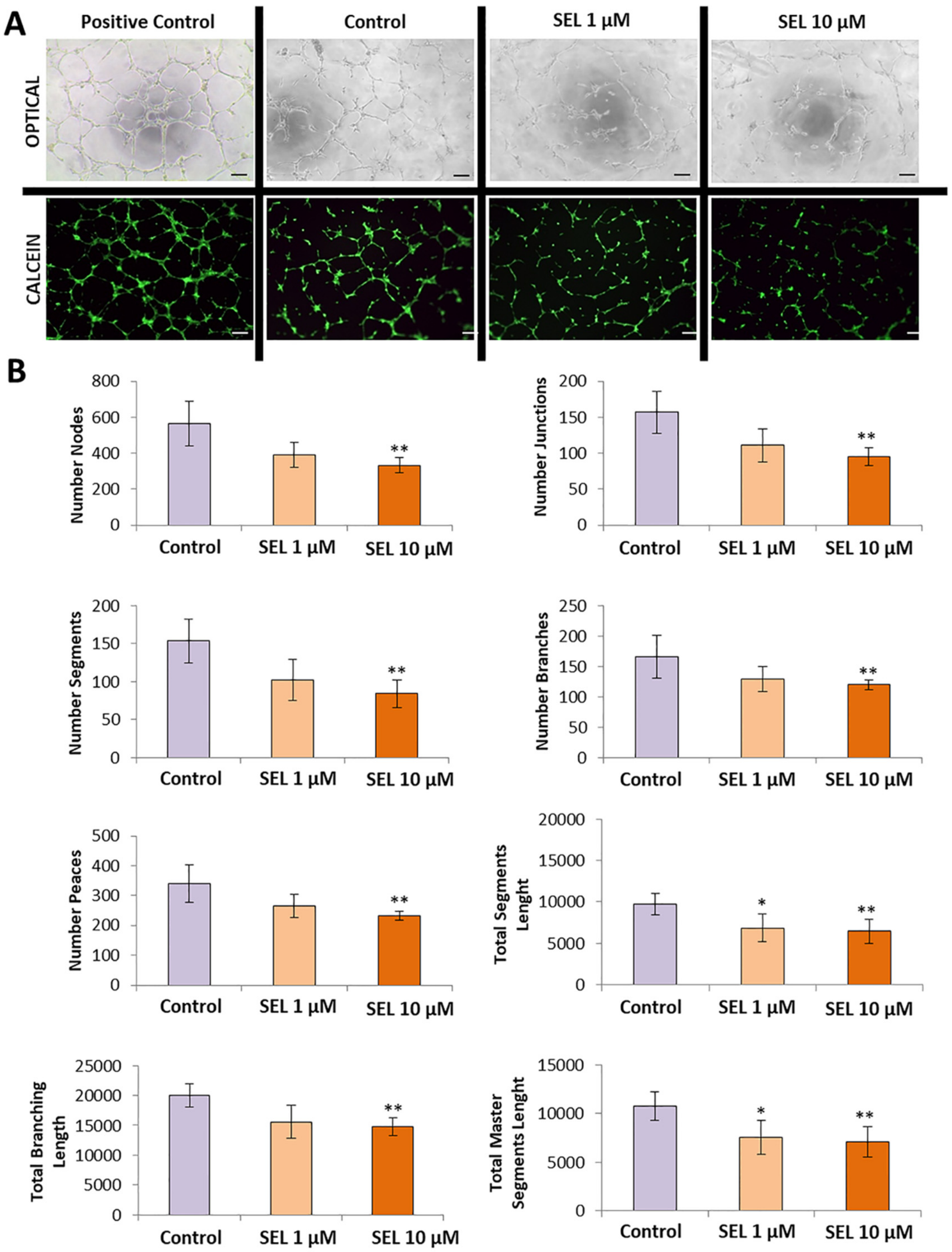

Figure S1. Effect of pancreatic cancer cells-conditioned media after sodium selenite treatment on angiogenesis. (**A**) Representative optical microscopy and calcein images of HUVECs exposed to conditioned media obtained from pancreatic cancer cells treated with sodium selenite (1 and 10 mM). (**B**) Graphical representation of different angiogenesis parameters measured on the calcein images including formation of nodes, branches, segments or junctions. From what can be seen, selenite at a dose of 10 clearly inhibits angiogenesis in HUVEC cells. Data are presented as the mean ± standard deviation of three independent experiments; * $P < 0.05$ and ** $P < 0.01$ vs. respective control group.
